# Automatic, global registration in laparoscopic liver surgery

**DOI:** 10.1007/s11548-021-02518-7

**Published:** 2021-10-26

**Authors:** Bongjin Koo, Maria R. Robu, Moustafa Allam, Micha Pfeiffer, Stephen Thompson, Kurinchi Gurusamy, Brian Davidson, Stefanie Speidel, David Hawkes, Danail Stoyanov, Matthew J. Clarkson

**Affiliations:** 1 Wellcome/EPSRC Centre for Interventional and Surgical Sciences and the Centre for Medical Image Computing, UCL, London, UK; 2 Division of Surgery and Interventional Science, UCL, London, UK; 3 Translational Surgical Oncology, National Center for Tumor Diseases, Dresden, Germany

**Keywords:** Deep learning, Semantic contour detection, Image guidance, Augmented reality, Laparoscopy, Automatic registration

## Abstract

**Purpose:**

The initial registration of a 3D pre-operative CT model to a 2D laparoscopic video image in augmented reality systems for liver surgery needs to be fast, intuitive to perform and with minimal interruptions to the surgical intervention. Several recent methods have focussed on using easily recognisable landmarks across modalities. However, these methods still need manual annotation or manual alignment. We propose a novel, fully automatic pipeline for 3D–2D global registration in laparoscopic liver interventions.

**Methods:**

Firstly, we train a fully convolutional network for the semantic detection of liver contours in laparoscopic images. Secondly, we propose a novel contour-based global registration algorithm to estimate the camera pose without any manual input during surgery. The contours used are the anterior ridge and the silhouette of the liver.

**Results:**

We show excellent generalisation of the semantic contour detection on test data from 8 clinical cases. In quantitative experiments, the proposed contour-based registration can successfully estimate a global alignment with as little as 30% of the liver surface, a visibility ratio which is characteristic of laparoscopic interventions. Moreover, the proposed pipeline showed very promising results in clinical data from 5 laparoscopic interventions.

**Conclusions:**

Our proposed automatic global registration could make augmented reality systems more intuitive and usable for surgeons and easier to translate to operating rooms. Yet, as the liver is deformed significantly during surgery, it will be very beneficial to incorporate deformation into our method for more accurate registration.

## Introduction

Augmented reality (AR) systems in laparoscopic liver surgery could help surgeons identify internal anatomical structures more clearly, especially in complex interventions. Such guidance can potentially reduce the risk of complications for the patients through safer decisions, reduced surgery time and less blood loss. An essential component in an AR system is the registration of the pre-operative 3D liver model and the intra-operative scene in the initial stage of the intervention.

Such registration presents several challenges since the liver undergoes significant deformation due to pneumoperitoneum, it is only partially visible and it lacks reliable features [8]. Most registration algorithms can be split into two stages. Firstly, a rough global rigid transform is estimated w.r.t. the laparoscopic scene. Secondly, local alignment methods improve the results further. Multiple automatic solutions have been proposed for local alignment, assuming a good initialisation [2, 18, 20]. For liver surgery, global alignment is usually achieved manually [21, 27, 29] or in a semi-automatic way requiring annotations from the clinician during the intervention [16, 26].

An automatic registration pipeline would remove any user induced variability. It could also be repeatedly employed in order to re-initialise the registration in case of occlusion (i.e. due to instruments, blood) without any additional load on the clinician. Global alignment is currently the main bottleneck in automatic long-term AR systems since subsequent local registration or tracking relies on this initial stage.

While stereo laparoscopes are used mostly in robotic systems, monocular scopes are much more commonly available. As such, the rest of the paper focusses on formulating a generally applicable approach, using a single 2D laparoscopic image.

## Related works

3D–2D liver registration methods require correspondences to be found between a 3D model and a 2D image of the patient’s anatomy. The main sources of failure in the alignment estimation stem from the deformation and the partial visibility of the liver in the laparoscopic image. To tackle these challenges, prior information can be used to constrain the optimisation. The liver boundary [2], the anterior ridge and falciform ligament [15, 16, 20, 26] have been proposed as landmarks for constraints. Currently, these techniques need manual annotation during surgery in order to obtain the liver contours [2, 15, 20], matching endpoints [15, 20] or manual global alignment [2]. While the annotation of the liver boundary could be automated using deep learning [13], a rigid initialisation of the camera pose is still needed, which is currently achieved manually [2]. Separating the organ boundary into the anterior ridge and silhouette can lead to automating the registration since they can be matched to the corresponding contours on the 3D model [15], if a large part of both contours is visible in the image. Several approaches have been validated in synthetic experiments for partial data assuming 70–100% of the liver boundary is visible [2, 20]. While such visibility ratios are achievable in open surgery [1], having an unobstructed large view of both liver lobes in laparoscopic intervention requires the cutting of the falciform ligament [15, 20]. However, laparoscopic images from interventions where the falciform ligament is present show only approximately 30–50% of the liver boundary. For such cases, manual alignment is currently the only reliable option.

An alternative with promising results in the computer vision research consists of using deep learning techniques to deal with the complexity of 3D–2D registration. Such techniques have been proposed in the medical field for clinical applications where standardised datasets can be collected easily, i.e. MRI, CT, OCT scans [3]. In image guidance, such 3D–2D registration datasets are not currently available and they are extremely difficult to build. As each surgery is slightly different in pathology, organ appearance, organ geometry, patient age, type of intervention, a large dataset with multiple examples for each task is needed. Since liver surgeries are especially challenging in terms of inter-subject variability, small datasets will result in the trained network being incapable of generalising well to new examples. For instance, a liver segmentation network trained on approximately 2000 images across 13 interventions reported poor generalisation with cases that vary too much from the training data [13].

While collecting more data requires extensive manual work and data collection from multiple surgeries, a solution could be provided by training networks with synthetic data. Several approaches propose to use synthetic data for CNN-based deformation estimation from partial surfaces in 3D–3D registration [5, 23]. However, building a completely simulated dataset for 3D–2D registration is extremely challenging, due to the domain gap between synthetic and real clinical data.

Recent studies propose style transfer to enhance the realism of surgical simulations [17, 22]. Specifically, a synthetic dataset of photorealistic simulations of laparoscopic liver surgery is publicly available^1^ [22]. Such large synthetic datasets are essential for advancing the current state of the art, but the issue of automatic 3D–2D registration is still not solved.

Alternatively, deep learning techniques can be used for solving well-defined tasks as part of a pipeline, such as contour detection. In the computer vision community, a real-time 3D eyelid tracking from semantic edges approach is most similar to our work [30]. They use a CNN to detect four edges of the eyelid, namely the double-fold, upper eyelid, lower eyelid and lower boundary of the bulge. These detected contours are then used to reconstruct the 3D shape and motion of the eyelids with increased realistic detail. While some similarities do exist, their method assumes the whole eye is visible at all times and explicitly uses the intersection points at the endpoints of the eyelid in the registration formulation. In laparoscopic liver surgery, such an approach would not be possible due to the partial visibility of the organ. Moreover, the variety of liver appearance and illumination fall-off due to the light source being close to the surface make the laparoscopic environment more complex. François et al. [11] propose a CNN-based framework to detect occluding contour of uterus. Occluding contours refer to boundary regions where the uterus occludes other structures and they are thus a subset of the uterus’s silhouette. This is in contrast to our method which detects the ridge as well as silhouette. This difference arises from the anatomical difference between the liver and uterus where the liver has a distinctive ridge region, but the uterus has a general spherical shape without outstanding features.

**Fig. 1:**
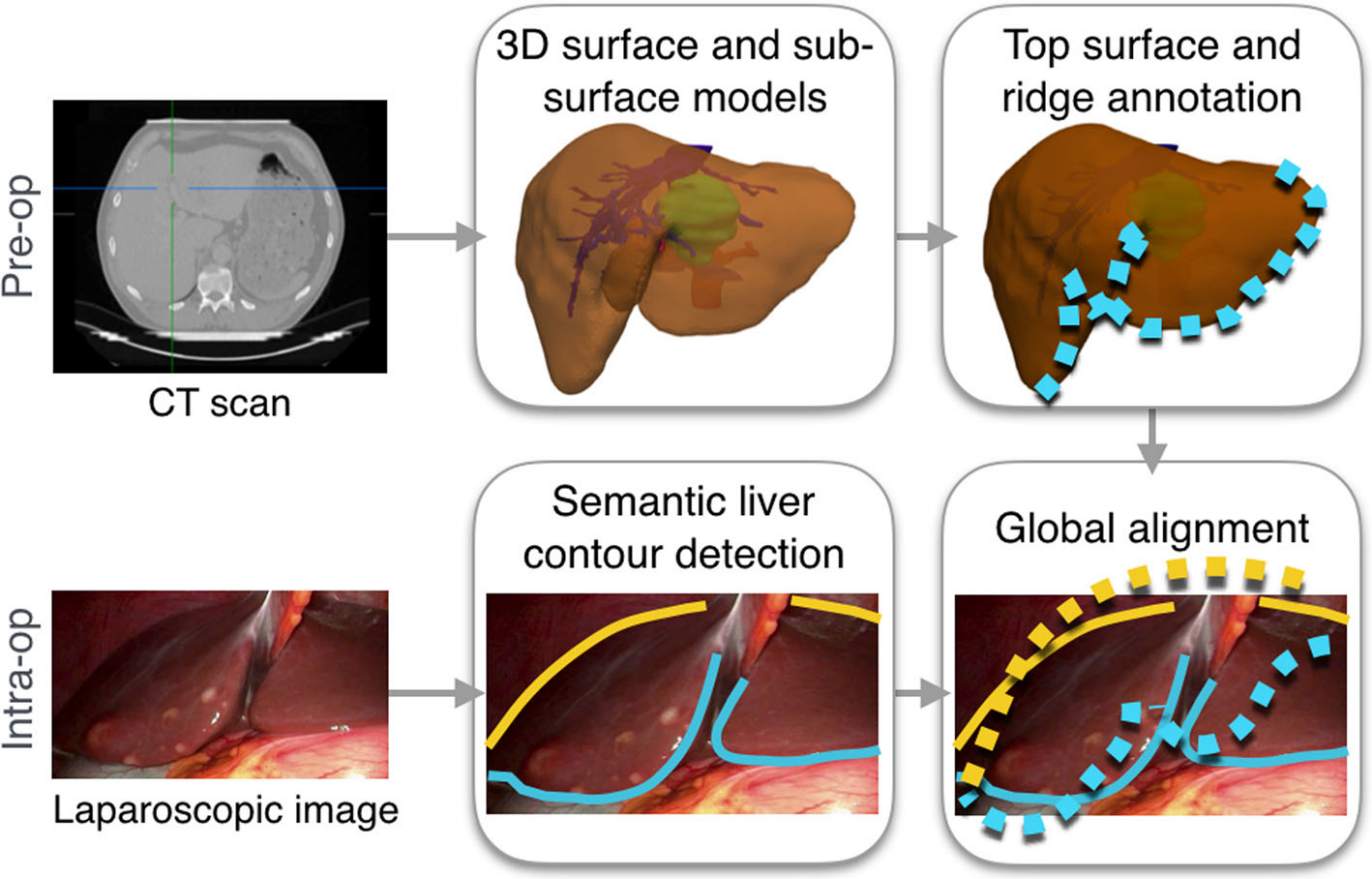
An overview of the proposed pre-operative and intra-operative stages for a global 3D–2D registration. Colour coding: silhouette—yellow, anterior ridge—blue

## Contributions

We propose an automatic global 3D–2D registration solution for general laparoscopic liver interventions. This work follows from a body of work utilising contours for 3D–2D liver registration [15, 16, 20, 26], to specifically address full automation. We have developed an automated contour detection algorithm that requires no manual annotations, followed by registration. This enables fully automatic 3D–2D registration. A concurrent work [11] attempts the same goal for the surgery on the uterus.

Concretely, our contributions are as follows: Firstly, a semantic edge detection network is adapted to distinguish between different types of liver contours. Secondly, a traditional pose estimation technique is extended to match corresponding contours, which are only partially visible. We perform quantitative and qualitative experiments to assess the feasibility of the proposed method, which show promising results.

## Methods

An overview of the proposed workflow is shown in Fig. 1. The 3D surface and internal anatomical structures are segmented via a commercial service.^2^ We also take advantage of no time limit in the pre-operative stage to pre-compute the anterior ridge and the top surface of the liver from the segmented liver mesh. These steps could be easily automated as well [24], but we chose to do it manually due to the variety in liver surface geometry when there are abnormalities present. Moreover, the intrinsic parameters of the laparoscopic camera can also be estimated pre-operatively [32].

During surgery, there are two main components after the laparoscopic image to be registered is selected: (i) semantic liver contour detection; (ii) global 3D–2D contour-based registration. We propose to use two types of liver contours: the *anterior ridge* and *silhouette* (Fig. 1). The former is an anatomical landmark which remains fixed on the organ but can become occluded due to blood, fat or overlapping organs such as the bowel. Note that it is easy to move the liver to reveal the ridge when the bowel overlaps. The latter changes depending on the camera position and organ deformation. When used together, these contours can provide complementary constraints to the pose optimisation [15], which becomes essential under partial visibility.

### Semantic contour detection network

In the computer vision research community, the most recent approaches proposed for semantic edge detection use CNNs to achieve state-of-the-art results. We adapt CASENet [31] to predict silhouette and ridge contours of the liver, as well as background (i.e. non-liver pixels), from laparoscopic images. In addition, we pre-train the network on around 100,000 synthetic laparoscopic images because the size of our clinical dataset is very small, i.e. 133 images. This greatly helps address the overfitting on a small dataset as well as improve the generalisation capability of the network.

Once the anterior ridge and silhouette are predicted on the input laparoscopic image, they need to be matched to the corresponding contours on the pre-operative 3D liver model.

### 3D–2D contour-based registration

Solutions for camera pose estimation from known 3D–2D correspondences can be obtained using well-established computer vision techniques such as Perspective-n-Point (PnP) [19]. Random Sample Consensus (RANSAC) has been proposed to deal with outliers in the correspondence set [10]. A combined PnP-RANSAC approach has been used successfully in multiple AR applications due to its computational efficiency and robustness [19].
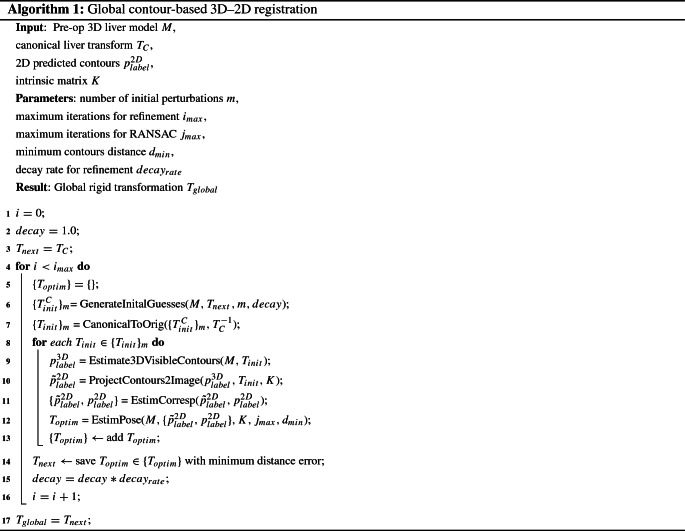


Algorithm 1 describes our proposed contour-based PnP-RANSAC extension. A transformation of the liver to a canonical space can be pre-computed ($T_{C}$), employing the common assumption that the laparoscopic camera will be inserted through a trocar placed approximately around the belly button of the patient [15]. Let the camera follow the right-handed coordinate system with the positive *x*- and *y*-axes pointing right and down, while the positive *z*-axis is pointing forwards. A range of *m* initial camera poses is generated in the canonical space $\{T^{C}_{init}\}_m$ by random perturbations of rotation around the *x*-, *y*- and *z*-axes ($\in \mathcal {N}(0,20^{\circ })$) of the camera (line 6), which makes the registration robust to smaller areas of the liver being visible. These initial transformation guesses $\{T^{C}_{init}\}_m$ are brought back to the original space of the 3D liver model (line 7). For each initial transformation guess (line 8), the visible contours are estimated on the 3D model (line 9) for each label, i.e. anterior ridge and silhouette. Firstly, the visible surface $M_{vis}$ is estimated from a given camera position (similar to [2]) by selecting the 3D liver model faces whose normal vector’s direction is within $\pm \,90^{\circ }$ of the vector from the centre of the face to the camera position. Secondly, the visible surface $M_{vis}$ is intersected with the top liver surface $M_{top}$. This step ensures all the points on the posterior side of the liver are excluded, since they are generally not visible in the initial stages of a laparoscopic intervention. Thirdly, the final 3D ridge points are obtained from the visible surface by $p^{3D}_\mathrm{ridge}$ = $M_{vis} \cap M_{ridge}$ where $M_\mathrm{ridge}$ is the pre-computed ridge surface. The reason why we separate the ridge points from $M_{vis}$ is that in some cases the top liver surface $M_{top}$ does not fully contain $M_{ridge}$, i.e. the ridge line sticks out of $M_{top}$, depending on the annotation. Therefore, we perform $p^{3D}_{ridge}$ = $M_{vis} \cap M_{ridge}$ to remove the unwanted portion of the ridge line for consistency. Lastly, the silhouette $p^{3D}_{silhouette}$ is estimated as all the boundary points of the visible surface $M_{vis}$ that do not belong to the ridge. Once the visible 3D ridge and silhouette points are estimated, they can be projected on top of the 2D image, using the known intrinsic parameters (line 10). Correspondences between the projected and predicted contours are computed in the image space by searching for the closest neighbour and similar normals (less than $30^{\circ }$ difference). The threshold used for normal similarity is to filter out correspondences where the projected and predicted contour shapes look different, even if the corresponding points are close in position, due to the deformation existing on the liver in the laparoscopic image [2]. As such, the function *EstimCorresp* on line 11 outputs a set of corresponding points $\{\tilde{p}^{2D}_{label},p^{2D}_{label}\}$ where the points $\tilde{p}^{2D}_\mathrm{label}$ belong to the projected 3D contours and the points $p^{2D}_{label}$ to the predicted contours on the laparoscopic image.

The PnP-RANSAC algorithm is then employed to find the optimal camera pose $T_{optim}$ for the current iteration (line 12). The PnP-RANSAC workflow consists of randomly selecting a minimal sample of 4 pairs from the correspondence set $\{\tilde{p}^{2D}_{label},p^{2D}_{label}\}$ at each iteration *j*. PnP is then employed to estimate the camera pose $T_{k}$ for the current minimal set of point pairs. We use the P3P technique proposed in [12]. In order to measure the agreement of the whole set of correspondences with the current estimate, a distance error is computed between the projected 3D contours (transformed using $T_{k}$) and the 2D contours. The chosen error is the modified Hausdorff distance [9] which enforces the corresponding contours to be similar. Concretely, the modified Hausdorff distance between sets $\mathcal {X}, \mathcal {Y} \subset \mathbb {R}^{n \times 2}$ is computed as1where $\mathbf {d}(a,\mathcal {B})$ is the minimum Euclidean distance between an element $a \in \mathbb {R}^{n \times 2}$ and a set $\mathcal {B} \subset \mathbb {R}^{n \times 2}$. We compute the modified Hausdorff distance separately for the ridges and the silhouettes as we empirically found that computing the distances separately yields better registration than computing the distance together, i.e. the ridge and silhouette contours are regarded as one contour. The final distance is the sum of distances of ridge and silhouette contours. Then, the optimal camera pose is the one with the minimum distance.

On top of this PnP-RANSAC loop (lines 8–13), we introduce another loop for refinement lasting $i_{max}$ iterations (line 4). This is to refine the estimated transformation further by starting a PnP-RANSAC loop with the optimal transformation from the previous iteration. After finishing the refinement loop, the global 3D–2D transformation $T_{global}$ is obtained.

The U-Net-based contour extraction and PnP-RANSAC-based registration are implemented within *SmartLiver* [27, 29], a closed source application for image guided liver surgery built on top of the open-source SciKit-Surgery libraries [28].

## Experiments

### Semantic contour detection

The training clinical dataset (C) consists of 133 images extracted from two laparoscopic interventions. The source videos were recorded using NifTK’s [6] IGIDataSources plugin. The data were annotated by a clinical fellow where polygonal lines were drawn on top of each contour type. The pre-training dataset (S) consists of approximately 100,000 synthetic laparoscopic images generated using [22].

**Fig. 2:**
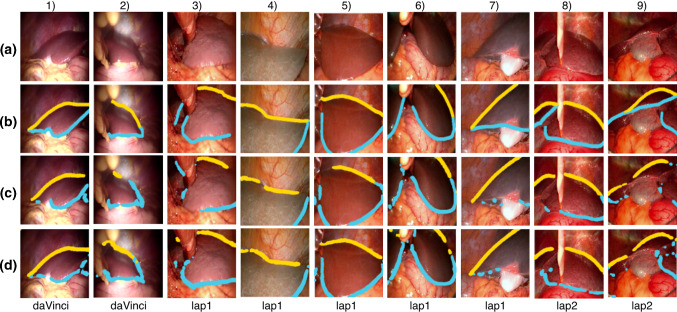
Qualitative results of ridge (blue) and silhouette (yellow) prediction. **a** Original images from the specified test dataset; **b** ground truth annotations; **c** predictions when CASENet was trained only on a clinical dataset (I+C); **d** predictions when CASENet was trained on synthetic and clinical dataset (I+S+C)

Two training scenarios are considered, where the weights for the CASENet model are first initialised from ResNet50 pre-trained on the ImageNet dataset (I) [7]: (i) **I + C**: CASENet is trained on the clinical dataset (C); (ii) **I + S + C**: CASENet is pre-trained on the synthetic dataset (S), then fine-tuned on the clinical dataset (C). Data augmentation is used to make the network invariant to brightness changes, contrast, rotations, translations, scale changes and shear. When pre-training CASENet on the synthetic dataset, we use as data augmentation only brightness changes and contrast in order to make the predictions more insensitive to different liver appearances. The Adam optimiser [14] was used for training the network with learning rate $1 \cdot e^{-4}$, and the training lasts for 300 epochs. A checkpoint is saved at the lowest validation loss, which is used to generate the results presented here. Train/validation set split is 80%/20%, respectively. The computation time for prediction on an image (using an NVIDIA GeForce GTX 1060 card) is around 140 milliseconds which is acceptable for use during surgery. We evaluate the performance of the proposed model on 3 test datasets: *daVinci*—9 images from a da Vinci intervention; *lap1*—9 images from 6 clinical cases; *lap2*—12 images from 1 clinical case. Figure 2 shows a selection of images from each dataset with the ground truth annotations and the predictions obtained using the two training scenarios.

**Table 1:** Performance of CASENet compared against a baseline, U-Net, on the test datasets measured by $F_1$ score ($F_1$), recall (*R*) and precision (*P*)

	Dataset		daVinci		lap1		lap2	
	Training		I+C	I+S+C	I+C	I+S+C	I+C	I+S+C
U-Net	$\mathrm{F_1}$	*Ridge*	0.37	0.43	0.37	0.41	0.24	0.54
		*Silhouette*	0.62	0.73	0.74	0.75	0.75	0.74
	R	*Ridge*	0.38	0.54	0.35	0.48	0.15	0.44
		*Silhouette*	0.74	0.84	0.73	0.82	0.71	0.83
	P	*Ridge*	0.39	0.38	0.43	0.37	0.72	0.70
		*Silhouette*	0.55	0.64	0.77	0.70	0.81	0.69
CASENet (ours)	$\mathrm{F_1}$	*Ridge*	**0.39**	**0.46**	**0.41**	**0.45**	**0.29**	0.42
		*Silhouette*	0.61	**0.75**	0.74	**0.76**	0.73	**0.77**
	R	*Ridge*	**0.39**	0.46	**0.42**	**0.51**	**0.20**	0.32
		*Silhouette*	0.57	0.79	**0.74**	0.82	0.68	0.77
	P	*Ridge*	**0.43**	**0.48**	**0.44**	**0.45**	0.63	0.66
		*Silhouette*	**0.70**	**0.71**	0.75	**0.72**	**0.82**	**0.79**

The numbers represent the average over all the images in each dataset. Higher numbers are better. (Bold numbers are when our method performs better than the baseline.) Notice that using the synthetic dataset (I+S+C) boosts the performance

**Fig. 3:**
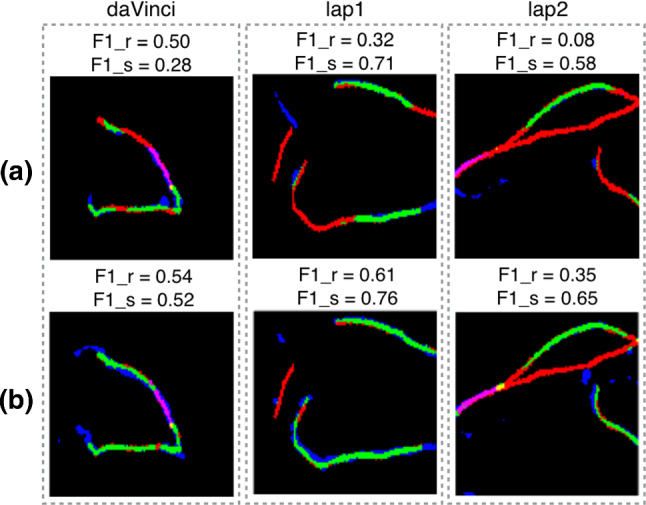
Example of $\mathrm{F_1}$ score maps for the ridge ($\mathrm{F1}\_\mathrm{r}$) and silhouette ($\mathrm{F}1\_\mathrm{s}$) where green $=$ true positives, blue $=$ false positives and red $=$ false negatives. **a** Predictions when CASENet was trained on the clinical dataset ($\mathrm{I+C}$); **b** predictions when CASENet was pre-trained on the synthetic dataset and fine-tuned on the clinical one (I+S+C)

Three accuracy measures are used for evaluating the network performance: *precision* P (out of all the predicted contour pixels, how many are correctly labelled?), *recall* R (how many of the ground truth contour pixels are predicted as correct?) and $\mathrm{F_1}$ score [4] which is defined as2where $\epsilon$ is a small number to avoid the denominator being zero. Table 1 summarises the results for each dataset in the two training scenarios using modified CASENet (ours) and a baseline method, U-Net. Figure 3 shows quantitative maps with true positives (green), false positives (blue) and false negatives (red) for some of the predictions in the test datasets, along with their associated $\mathrm{F_1}$ scores.

### Quantitative experiments

The registration performance depends on the uniqueness of the constraints imposed by the contours. Since the characteristics of the contours (such as the curvature) vary greatly depending on the viewing angle, both partial visibility and the liver region need to be taken into consideration.

We adapt the pre-operative simulation framework proposed in [25] for quantitatively analysing the performance of the proposed 3D–2D contour-based registration. Originally, the method in [25] was used for pre-operatively computing a data acquisition protocol in which the surgeon would acquire specific liver surface patches which would ultimately lead to an efficient 3D–3D registration. Their approach is appealing because it provides a way to analyse which specific camera views would result in a good registration.

**Fig. 4:**
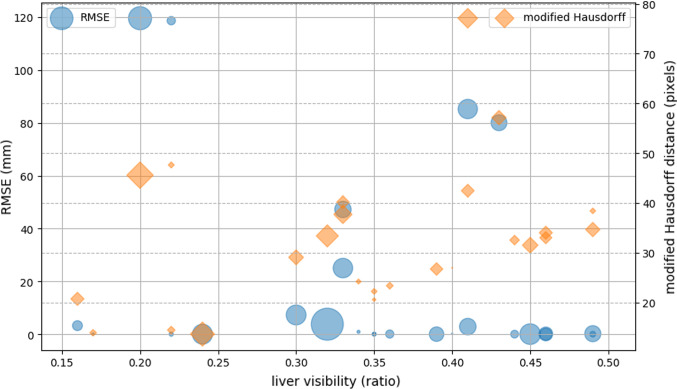
The registration errors (RMSEs) on the synthetic dataset along with the modified Hausdorff distances against visibility of the liver. The modified Hausdorff distance is what our method minimises for. For each trial, a synthetic image is generated from a random camera pose. Then, a 3D liver surface is registered to the image 10 times to compute the median and standard deviation (std) of the modified Hausdorff distance and RMSE. The area of each circle/diamond is proportional to the std. (Largest area corresponds to std of 151.30 for RMSE and 51.81 for modified Hausdorff distance.) The initial RMSEs, computed at the beginning of each registration, throughout the experiments are around 250 mm. Note that the failed registrations with high RMSEs ($> 45$) have less than 30% (0.30 in the figure) liver, ridge or silhouette visibility (ridge and silhouette visibilities not shown in the figure)

The simulation framework loads a 3D liver model from a clinical case. In our synthetic experiments, 25 random camera positions are simulated on a sphere around the liver. The camera orientation is perturbed further in order for the liver not to be always at the centre of the image. For each camera, a synthetic image is obtained by estimating the visible contours and projecting them to 2D. For each camera position, the proposed contour-based registration is run 10 times between the synthetic image and the 3D liver surface, in order to account for the sources of randomness (lines 6 and 12 in Algorithm 1). Figure 4 shows the results. The liver visibility was computed as a ratio between the visible front liver vertices over the total vertices of the front liver surface. The root-mean-square error (RMSE) is measured between the ground truth vertex positions of the 3D liver surface and the estimated vertices obtained after the registration process.

On top of analysing the robustness to partial visibility, such a pre-operative planning simulation can provide a clear protocol to clinicians with regard to which portions of the liver provide sufficient constraints for the registration.

The registration takes less than 1 min which makes our method suitable for intra-operative use.

### Qualitative experiments

**Fig. 5:**
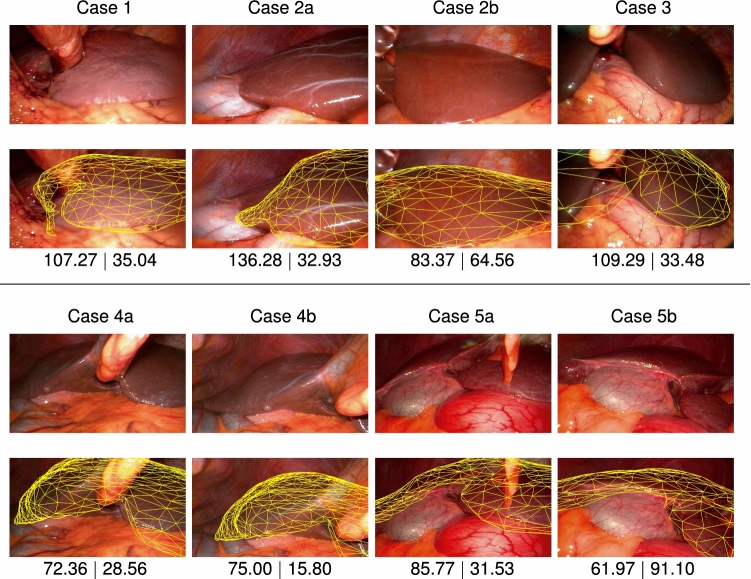
Results of the proposed global registration pipeline on 5 retrospective clinical cases. The first row for each case shows the input laparoscopic image and second row the registered 3D liver model overlaid on the image. The numbers on the bottom row are the reprojection error in pixels (on the left) and RMSE in millimetre (on the right). The reprojection error is computed by the modified Hausdorff distance between the ground truth contours and the projected contours of the 3D liver model. RMSE is computed against the manually registered liver model’s vertices

We perform experiments on real clinical data to assess the feasibility of our method in a laparoscopic liver intervention. The dataset used to validate the proposed registration pipeline is composed of 14 images from 5 retrospective clinical cases. Figure 5 illustrates the registration results where the 3D liver surface is overlaid on the input image. Without ground truth datasets for registration, we provide errors computed on the contours as well as on the vertices of the liver model against manually registered liver model. For the contours, the modified Hausdorff distance between the ground truth contours and the projected contours of the 3D liver model is computed. For RMSE, we manually register the liver model on each image and compute RMSE between all the vertices of the manually registered liver and those of the liver registered by our method. These results show the potential of our proposed registration pipeline on challenging laparoscopic images.

## Discussion

### Semantic contour detection

Table 1 shows how the use of synthetic dataset improves contour prediction (I+S+C). Compared to U-Net (baseline), CASENet (ours) performs better on our task and datasets. However, it is worth noting that the choice of the network architecture might not be the most critical factor for the better performance and other networks such as U-Net may suffice.

Figure 2 shows an excellent generalisation across livers with significant changes in appearance (i.e. columns 4, 6, 7) and across different image acquisition methods. Notice how pre-training improves how much of the contour gets detected, especially on the last column which presents a case never encountered in the training set C.

### Quantitative experiments

Figure 4 shows that the proposed registration method can cope with severe occlusion of the liver surface, thus occlusion of ridge and silhouette. It manages to estimate a good initial alignment (within several cm [18]) with as little as 30% visible front liver surface. Since laparoscopic images generally capture approximately 30–50% of the front liver surface, these results are highly encouraging. Notice that the failed registrations with high RMSEs ($> 45$) have less than 30% (0.3 in the figure) visibility in either liver, ridge or silhouette. (Ridge and silhouette visibilities are not shown.)

### Qualitative experiments

The proposed pipeline was successful in estimating a global alignment on all 5 clinical cases in the registration dataset. Figure 5 shows the registration results where the 3D liver surface is overlaid on each input image. As observed in the figure, the proposed pipeline achieves acceptable registration for the initial registration purpose on challenging laparoscopic images with various liver geometries, appearances and viewpoints. Still, it can be observed that the deformable registration will be highly beneficial to achieve more accurate registration as the intra-operative liver shape is significantly deformed from the pre-operative one.

## Conclusion

We propose a novel fully automatic pipeline to globally register a pre-operative 3D model to a single laparoscopic image during liver interventions. The first stage involves a semantic liver contour detection network which estimates the location of the anterior ridge and the silhouette. These contours are then matched with the ones on the pre-operative 3D model in order to estimate a global rigid registration.

Validations of the proposed pipeline were conducted on synthetic and clinical data. With the synthetic data, we show that the proposed registration can estimate a global alignment with as little as 30% of the liver surface visible by extending a patient-specific pre-operative analysis. Also, the proposed registration pipeline was successfully applied in 5 retrospective clinical cases and it was robust to the occasional errors in the contour prediction stage.

We hope the proposed automatic global registration pipeline can improve augmented reality systems in laparoscopic interventions to be more efficient and intuitive for surgeons.

## Footnotes

1 http://opencas.dkfz.de/image2image/.

2 www.visiblepatient.com.
